# The Sleep–Wake Cycle Pattern of a Blind Trail Ultramarathon Runner and His Guide: The World’s First Case

**DOI:** 10.3390/clockssleep7020020

**Published:** 2025-04-15

**Authors:** Larissa Quintão Guilherme, Julia Pagotto Matos, Ana Claudia Pelissari Kravchychyn, Marco Tulio De Mello, Paulo Roberto dos Santos Amorim, Helton de Sá Souza

**Affiliations:** 1 Department of Physical Education, Federal University of Viçosa (UFV), Viçosa 36570-900, Brazil; larissa.guilherme@ufv.br (L.Q.G.); julia.pagotto@ufv.br (J.P.M.); pramorim@ufv.br (P.R.d.S.A.); 2 Department of Nutrition, Federal University of Viçosa (UFV), Viçosa 36570-900, Brazil; ana.pelissari@ufv.br; 3 Department of Sports, Federal University of Minas Gerais (UFMG), Belo Horizonte 31270-901, Brazil; tmello@demello.net.br; 4 Faculty of Health Sciences, Universidad Autónoma de Chile, Providencia 7500912, Chile; 5 Research Center on Motor Activities (CRAM), University of Catania, 95124 Catania, Italy

**Keywords:** visually impaired, ultramarathon running, sleep, circadian rhythm, physical performance

## Abstract

Trail running has seen a surge in participants, including individuals with disabilities, particularly in ultratrail running (UTRs). Sleep–wake patterns are crucial for optimal performances in UTRs, which present unique physiological and behavioral challenges. This case study evaluated the sleep–wake cycle of a blind trail ultramarathoner (BTR) and his guide (GTR) before, during, and after an 80 km UTR. Two male participants (BTR: 54 years, BMI: 26.1 kg/m^2^; GTR: 48 years, BMI: 24.2 kg/m^2^) were assessed using validated questionnaires (MEQ, ESS, ISI, and PSQI) and actigraphy over 35 days. The BTR exhibited a morning chronotype (MEQ = 63), mild insomnia (ISI = 11), poor sleep quality (PSQI = 5), and prolonged sleep latency (>60 min), while the GTR showed an indifferent chronotype (MEQ = 52), good sleep quality (PSQI = 3), and shorter latency (10 min). Post-competition, both athletes experienced an increased total sleep time (TST): the BTR by 17.8% (05:32:00 vs. 04:25:00) and the GTR by 5.5% (07:01:00 vs. 06:39:00). The BTR demonstrated a greater Wakefulness after sleep onset (WASO 01:00:00 vs. 00:49:00) and awakenings (15.4 vs. 6.1). A time series analysis revealed greater variability in the BTR’s post-competition sleep efficiency and TST, while the GTR exhibited a greater stability of the circadian phase. These findings highlight the intricate sleep challenges faced by blind athletes, informing strategies to optimize recovery and performance.

## 1. Introduction

Trail running has been gaining more prominence, with records of a substantial increase in participants [1]. Although there is no official competition specifically for people with disabilities, several organizers allow the inclusion of this population in their events. This increase in the number of participants with disabilities has occurred in races of all distances, especially in ultratrail running (UTR) events (>6 h duration).

The UTRs are pedestrian races that pass through mountains, deserts, glaciers, or any other environment. It is important to note that more than 80% of the route needs to consist of natural surfaces [2,3]. In this way, the physiological and behavioral challenges, due to the high volume and intensity of this type of competition, are extreme [3,4]. For a blind individual and his/her guide, this challenge is possibly even greater. To maintain an optimal performance in the face of the diversity of UTRs, runners need to keep some parameters in balance, including good nutritional management [2,5] and water intake [6,7] and the synchronization between waking, include training moments, and sleeping behaviors [5,8].

Adequate sleep plays a fundamental role in physical and cognitive restoration and can positively influence performance during and after competitions [9,10]. Contrary to this perspective, it is well established in the literature that exercises with high overloads, as is the case with UTRs, can harm a good balance between the factors mentioned above [8,10]. Specifically, regarding sleep, it is well established that blind individuals are more prone to sleep–wake cycle disorders [11,12] and major sleep disturbances [13,14]. In some types of blindness, light perception is absent, which compromises the alignment of the biological clock and leads to a greater chance of circadian rhythm desynchronization, such as phase delay disorder, and altered sleep patterns [14,15]. These changes, together, increase the odds of sleep disorders, such as insomnia and excessive daytime sleepiness [14,16], which can directly impact the athletes’ performance [8,17,18].

Along with the popularization of UTRs, it is possible to observe a constant increase in scientific production on the subject in the last decade [4,6,19,20,21]. However, as far as we know, little research has been carried out on the profile of runners with disabilities in this modality, as well as the effects of competition on this population and the sleep–wake cycle. Therefore, understanding the influences of sleep variables on outdoor sports performance, specifically for blind trail ultramarathon athletes, is at the forefront of scientific development. Thus, this is the first study that aims to demonstrate the sleep–wake cycle pattern of an ultramarathon trail runner with total visual impairment and his guide during the month before, during, and after an 80 km competition.

## 2. Results

### 2.1. Subjective Sleep Outcomes

The blind trail ultramarathon runner (BTR) was classified as a morning chronotype (H.O. score = 63), while the guide trail ultramarathon runner (GTR) had an indifferent chronotype (H.O. score = 52). Both the GTR and BTR had a high chance of sleepiness, but only the BTR had the characteristics of insomnia, even if mild (IGI score = 11), while the GTR does not (IGI score = 1). It was also observed that in the last 30 days before the competition, the BTR had self-reported a sleep quality classified as poor (PSQI score = 5 and sleep latency greater than 60 min). Despite this, the self-reported TST was 480 min, and the SE was calculated at 88.9%. In contrast, the GTR had a good sleep quality (PSQI score = 3), with a latency of 10 min, TST duration of 410 min per night, and SE of 97.6% (Table S1).

### 2.2. Objective Sleep Outcomes

Individual sleep patterns compared on weekdays and weekends are detailed in the Supplementary Tables S2 and S3, while comparisons of pre- and post-competition means are in Table S4. It was observed that during weekdays (Table S2), the GTR maintained greater regularity in pre-race bedtimes and went to bed earlier in the post-race week. Both maintained some regularity in their wake-up time, with the BTR waking up later in the post-race week (07:59:00 ± 03:25:00). In contrast, the GTR woke up earlier in that week (06:14:00 ± 00:15:00). The BTR spent more time in bed after the race (07:19:00), while in the week before the race, this time was shorter (05:49:00), resulting in less TST in this period (∆ = 01:07:00, 17.80%). The GTR, in turn, spent more time in bed after the race (07:55:00 ± 01:39:00) and slept more in this week (07:01:00 ± 01:25:00).

The BTR’s sleep latency varied between 4 and 6 min, while the GTR showed a higher latency (00:17:00). Both maintained good sleep efficiency (BTR = 88.46 ± 2.17 vs. GTR = 88.33 ± 1.90). Regarding the wakefulness after sleep onset (WASO), in the post-race week, an increase was observed in the BTR (01:00:00, ∆ = 00:11:00, 18.30%), with more awakenings also recorded (15.40 ± 6.15). The GTR demonstrated a higher WASO between the pre- and post-race week (00:50:00 ± 00:32:00 and 00:49:00 ± 00:26:00).

During the weekends, both tended to sleep and wake up later compared to their usual weekdays. Regarding the time in bed and TST, both presented higher times in the pre-race week and lower times in the post-race week. This resulted in a higher ESS on the weekend before the competition and a lower ESS after the race. These results are presented in Table S2.

**Figure 1:**
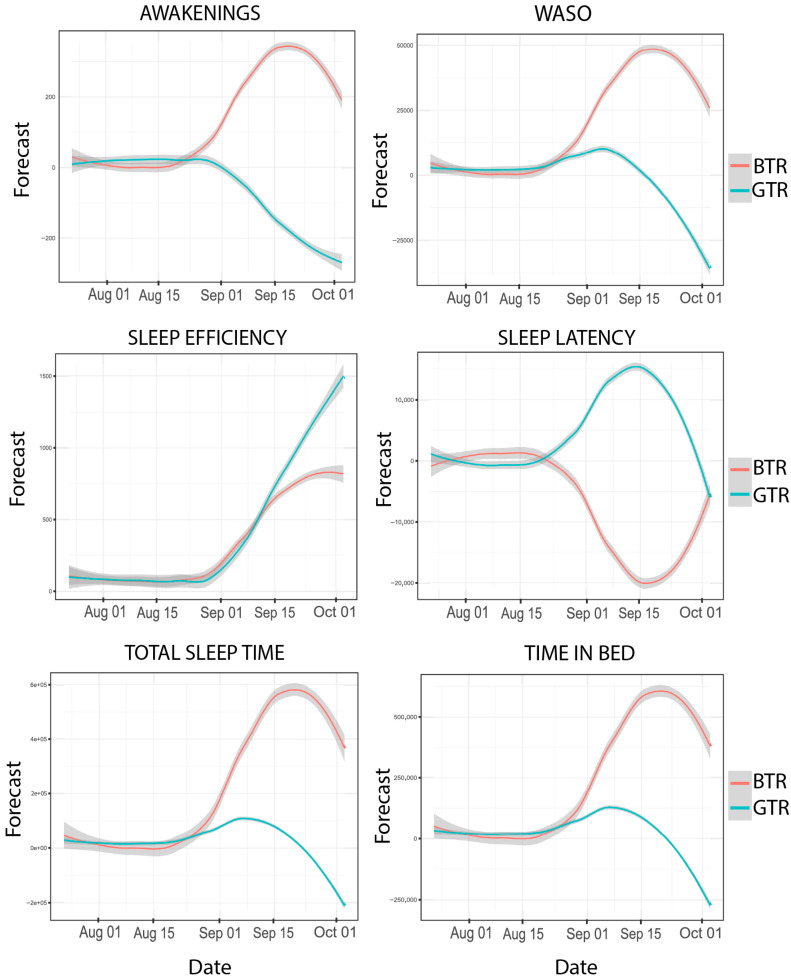
Graphical representation of sleep time series analyses. BTR = blind trail ultramarathon runner and GTR = guide trail ultramarathon runner.

These results become even more evident after analyzing the time series (Table 1 and Figure 1), as there were significant differences in bedtimes, with a significant, consistent variability, confirmed by the similar BIC between them (BTR_(BIC)_ = 20.8, *p* < 0.01 vs. GTR_(BIC)_ = 20.98, *p* = 0.03). For the time of waking up, the GTR showed a greater consistency in the time of waking up with a progressively lower BIC (GTR_(BIC)_ = 17.09, *p* = 0.03) in relation to the BTR (BTR_(BIC)_ = 17.73, *p* =0.66). Concerning the time in bed, the BIC and RMSE indicated less variation for the GTR (GTR_(BIC)_ = 18.10, RMSE = 8072.47) than the BTR (BTR_(BIC)_ = 17.39, RMSE = 5655.01, *p* = 0.04). In the latency, consistent differences were observed between participants, with a close BIC for both (BTR_(BIC)_ = 10.77, *p* < 0.01 vs. GTR_(BIC)_: 10.54, *p* < 0.01). In terms of SE, there was significant variability, where the GTR obtained a higher BIC (3.86) and RMSE (6.54, *p* = 0.04) than the BTR (BTR_(BIC)_ = 2.60, RMSE = 3.48, *p* < 0.01). Finally, the number of awakenings was higher for the GTR (GTR_(BIC)_ = 4.74, RMSE = 10.10, *p* = 0.002) compared to the BTR (BTR_(BIC)_ = 3.26, RMSE = 4.84, *p* < 0.05).

### 2.3. Objective Circadian Rhythms Outcomes

In the analysis of the activity–rest cycle during weekdays (Table S5), the BTR demonstrated greater activity in the most active 10 h period (M10) in the third week (7164.62 counts), while the GTR exhibited greater activity in the week before the race (8276.36 counts). Both presented lower activity in the week following the competition (BTR = 3566.70 counts vs. GTR = 2016.99 counts).

On weekends, both presented the lowest M10 average in the second week (BTR = 2550.47 counts vs. GTR = 3142.42 counts) and the highest in the weekend before the competition (BTR = 9180.47 counts vs. GTR = 10,485.70 counts). The shortest 5 h least active period (L5) for both occurred in the pre-race weekend (BTR = 88.49 ± 0.58 vs. GTR = 91.95 ± 5.76). As for the mesor, both registered the lowest count in the second weekend (BTR = 1431.42 vs. GTR = 1803.18) and the highest in the first week (BTR = 5325.54 vs. GTR = 7106.74). The amplitude of the weekend days for the BTR varied from 694.97 to 5230.06, while that for the GTR was from 1803.18 to 7106.74. Finally, the acrophase of the BTR varied from −0.5245 to −5.7461 radians and that of the GTR from −2.6323 to −5.4367 radians.

When analyzing the averages of the activity–rest cycle during the weeks and weekends, a distinct dynamic is observed (Table S3). During the weeks, the BTR was 9.90% more active, as evidenced by the higher value of M10 (BTR = 5687.82 vs. GTR = 5128.19). However, on weekends this dynamic is reversed, indicating that the GTR spent 16.90% more time active than the BTR. Finally, on weekend days, in relation to pre-competition, there were no significant differences between the BTR and GTR data. However, on the post-competition weekend it was observed that the BTR had a smaller M10 (Δ = 1928.51 counts, 22.63%), acrophase (Δ = 3.891 radians, 88.12%), and mesor and a larger L5 (Δ = 584.36 counts, 62.47%) (Table S6).

**Figure 2:**
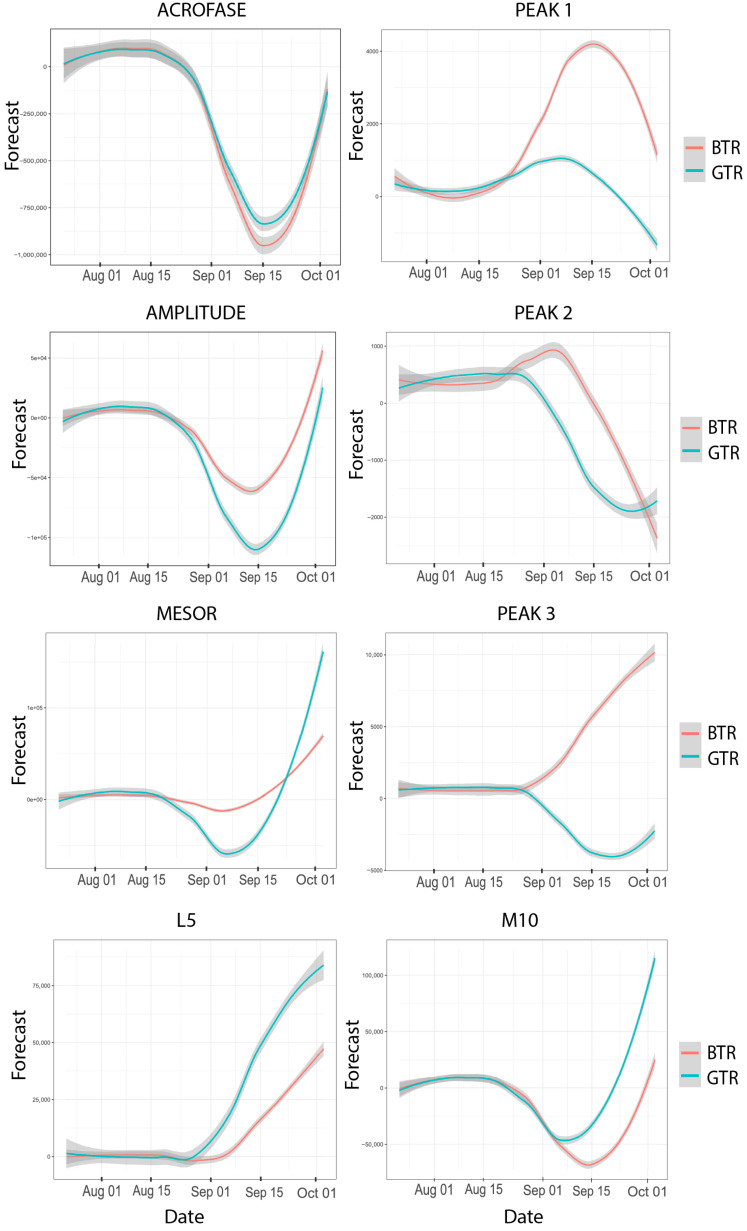
Graphical representation of time series analyses of circadian rhythm. BTR = blind trail ultramarathon runner and GTR = guide trail ultramarathon runner.

The results in Table 2 and Figure 2 show the data from the time series analyses between the BTR and GTR in relation to the circadian rhythm over the 35 days monitored. The models adjusted for the BTR showed less variability in the M10 (RMSE = 2998.08 vs. 4124.71) and L5 (RMSE = 1086.12 vs. 1275.10). Furthermore, the circadian amplitude was more consistent for BA (RMSE = 2489.47 vs. 3353.68). However, the acrophase values in radians were similar in the BIC and RMSE between both, although they were significant only for the GTR (*p* = 0.003), demonstrating greater stability in the phase of greatest activity of the GTR.

## 3. Discussion

To our knowledge, this is the first study that sought to analyze the sleep and activity–rest cycle of a trail ultramarathon blind athlete and his respective guide over 35 days (28 days) pre- and (7 days) post-competition. The results indicate that the main sleep of the GTR over the weeks was worse than that of the BTR, considering the parameters of latency and the number of awakenings. On the other hand, both the GTR and BTR presented good sleep efficiency. It was also the first time that it was demonstrated, based on the time series analysis technique, that the rhythmic and sleep desynchronization between the blind and guide athlete is high after the end of the competition. These results may suggest that the BTR’s elimination from the competition may be associated with sleep inadequacies during the competition preparation period.

When considering factors like sleep latency and the number of awakenings, the BTR’s sleep quality throughout the week was worse than that of the GTR. Additionally, both subjects consistently slept less than the recommended minimum of 7 h per night throughout the week [22] and even less on weekends prior to the competition. In addition, there is a higher likelihood of experiencing excessive daytime sleepiness. Copenhaver and Diamond [23] recommend that the sleep latency should be under 30 min and that the awake time after sleep onset should not exceed 20 min. Therefore, it is evident that sleep was impaired in the pre-competition condition. A decreased total sleep time is often linked to the stress associated with a competitive environment [10]. Additionally, athletes frequently experience sleep inadequacy due to their heavy training loads and the time allocated for training, which restricts their opportunities for sleep [24]. Bianchi, Miller, and Lastella [20] demonstrate similar results, indicating that during pre-competition conditions for a 326 km ultramarathon, the average TST was 6 h per night.

As previously mentioned, neither the BTR nor GTR completed the race, withdrawing at kilometer 40. The authors believe this situation may be linked to the athletes’ overall sleep quality. Both individuals slept less than necessary during the pre-competition period and exhibited a high likelihood of daytime sleepiness. This hypothesis is supported by temporal analyses, which revealed significant changes in the BTR’s sleep patterns following the race and contrasting changes in the guide’s sleep.

The temporal series data (total sleep time, time in bed, waking after sleep onset, sleep efficiency, sleep latency, and number of awakenings) and rhythm patterns (most active 10 h period, shortest 5 h least active period, acrophase, amplitude, peak 1, peak 2, and peak 3) demonstrate that the GTR and BTR differ after the competition period, as evidenced by the visual inspection presented in Figure 1 and Figure 2 of the ARIMA. This situation appears to be common among Paralympic athletes, who frequently encounter issues related to sleep quality and duration [25,26,27]. On the other hand, the circadian rhythms and sleep of these athletes were synchronized in the pre-competition period. Possibly, the desynchronized pattern, as observed after the competition, was also present at some point in the preparation process, though we were unable to identify it due to the time frame of data collection in this study.

The sleep patterns and circadian rhythm of the GTR and BTR may affect each other, forcing one of the athletes to seek to change their behaviors to prepare for the competition. Short-term changes in these behaviors can impact the decision-making processes [28,29,30,31], subjective perception of effort [32,33,34], and performance in endurance sports [10,19,29,34,35,36]. Therefore, it would be important that, in long-distance trail and mountain races, in addition to physical performance characteristics [19,35], blind athletes and guides also have similar sleep patterns and circadian rhythms [37].

A study involving 99 Paralympic athletes with visual impairments—competing in disciplines such as athletics, goalball, swimming, blind soccer, and judo—identified a high prevalence of sleep disorders, affecting approximately one-third of the athletes (32.1%) [15]. Furthermore, as noted by Roberts, Murphy, and Goosey-Tolfrey [25], visually impaired athletes frequently report difficulties falling asleep, frequent awakenings during the night, reduced total sleep time, and episodes of unintentional napping. These factors contribute to poor sleep quality, potentially impairing athletic performance.

The results of this study highlighted a possible rebound effect in sleep following the competition, as the BTR exhibited progressively poorer sleep over time. This trend was evident in the PSQI scores and objective sleep parameters predicted by the ARIMA, which indicated reduced sleep latency, increased WASO, and more frequent awakenings. These results suggest that sleep deprivation (partial or total) and accumulated sleep debt may influence cognitive function, daytime sleepiness, and athletic performance, as reported by O’Donnell et al. [38]. Additionally, Charest et al. [29] emphasized that even partial sleep deprivation can negatively affect cardiorespiratory and psychomotor performance, leading to faster exhaustion among athletes.

On weekdays, both individuals maintained consistent sleep and wake schedules; although, the BTR exhibited greater variability in certain aspects. However, on weekends both individuals tended to go to bed and wake up later than usual. This variability in sleep timing may be linked to a circadian rhythm resynchronization between the BTR and GTR as presented by the time series analysis. In visually impaired individuals, this desynchronization can occur due to the limited exposure to environmental light, which is essential for aligning the circadian pacemaker with the 24 h light–dark cycle. This can result in a progressive shift in sleep–wake patterns [39]. According to Atan et al. [14], blind individuals are more likely to experience symptoms such as insomnia and excessive daytime sleepiness due to circadian rhythm desynchronization, a phenomenon also evident in the BTR’s subjective results.

Regarding secondary sleep, the BTR napped for shorter durations than the GTR on weekdays but for longer durations on weekends. Nap efficiency was variable, being more consistent on weekends. Studies have highlighted the benefits of napping for athletes, showing that naps can improve physical and cognitive performance, particularly after partial sleep deprivation [38,40]. Furthermore, athletes often sleep fewer hours than required and tend to rely on naps to compensate for sleep loss, both on weekdays and weekends [24].

Finally, the findings of the present study indicate the need for strategies aimed at improving both the quality and quantity of sleep, as well as the circadian rhythm of visually impaired athletes, especially ultramarathon runners, to prevent potential future health and performance impairments. Silva et al. [41] demonstrated that, among Paralympic athletes, insomnia symptoms, frequent awakenings, and sleep fragmentation are positively correlated with more severe health problems. Therefore, we emphasize the importance of adopting strategies to minimize sleep-related impairments in the week leading up to a competition. These include implementing interventions to promote sleep extension, sleep hygiene, and stress reduction in the weeks prior to competition, with the aim of improving sleep quality and potentially enhancing athletic performance [8,25].

Given the results, it is important to acknowledge the limitation of this being a cross-sectional case study. Nonetheless, the need for further investigations into the sleep–wake cycle of blind athletes, particularly ultramarathon runners, is evident. Such research is crucial to identify and gain a deeper understanding of their rhythmic patterns. This knowledge is essential for developing strategies to preserve and improve sleep quality, thereby enhancing physical performance.

## 4. Materials and Methods

This study is an observational case study. All ethical precautions were taken in this study to ensure the integrity of the participants.

### 4.1. The Case

The sample consisted of a blind trail ultramarathon runner (BTR) and his guide (GTR); both had more than two years of experience in the sport and were finalists in the previous edition (2022) of this same race and were regularly registered to participate in the 80 km race of La Misión Brasil (La Misión 80K), held in 2023. The route has not changed between editions. Regarding the characteristics of the athletes (see Supplementary Table S1), both were male, but the BTR was 54 years old, with a height of 1.75 m tall and a BMI of 26.1 kg/m^2^, and the GTR was 48 years old, with a height of 1.69 m tall and a BMI of 24.2 kg/m^2^.

**Figure 3:**
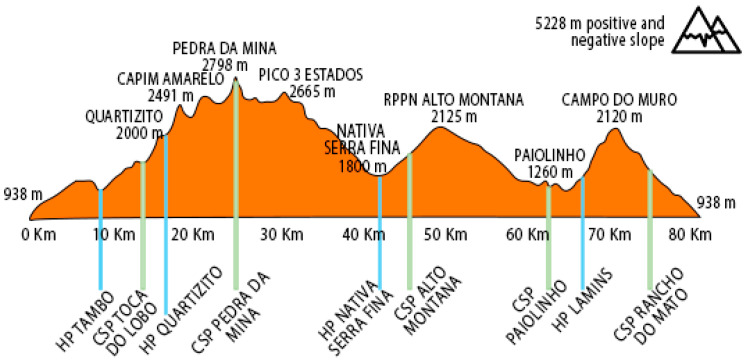
Altimetric maps and stands of feeding and hydration localization.

La Misión 80K is the most difficult trail run in Brazil. The route reaches 5288 m of elevation gain (altimetry), crossing the 4th highest peak in the country (2798 m altitude). In this case study, despite the planned route being 80 km, the athletes were disqualified after reaching 40 km because the BTR showed a significant drop in performance between the 2nd and 3rd control and support post (CSP), at the hydration point (HP Nativa Serra Fina). The GTR reported that he would be able to maintain his performance and that, if he were competing individually, he would continue his race. In the disqualification section, the athletes had already accumulated approximately 2665 m of altimetry (Figure 3).

### 4.2. Assessment Instruments

The Horne and Ostberg Morningness and Eveningness Questionnaire (MEQ) [42] was used to assess chronotypes; the Epworth Sleepiness Scale (ESS) was used to identify possible daytime sleepiness [43,44]; the Insomnia Severity Index (ISI) is capable of identifying difficulties in initiating or maintaining sleep, possible early awakenings, interference in routine activities and satisfaction with sleep [45]; and the Pittsburgh Sleep Quality Index (PSQI) was used to assess subjective perceptions of sleep [46].

For the objective monitoring of the sleep–wake cycle, an ActTrust 2 actimetry device (Condor Instruments, São Paulo, Brazil) was used, starting 28 days before the competition, during the competition, and continuing for 7 days post-competition. Data analysis was performed using ActStudio^®^ 2.2.3. software. The following data were assessed: bedtime; wake-up time; time in bed; total sleep time (TST); latency (the period between bedtime and the moment of sleep onset); sleep efficiency (SE); WASO (waking after sleep onset, time awake after the onset of the main sleep); and number of awakenings [47].

The circadian rhythm metrics between activity and rest, assessed by actimetry, included the average of the most active 10 h period (M10); the 5 least active hours (L5) [48]; the peaks; the time in which the variable reaches its maximum value (acrophase); the average value of the adjusted rhythm (mesor: Midline Estimating Statistic of Rhythm); and the difference between the mesor and the minimum and maximum value (amplitude) [49].

As recommended by De Moura Simim [50] and Fekedulegn et al. [51], concomitantly with the use of the actimetry, the athletes recorded information in a sleep diary during the same periods of use, providing data for the analysis of sleep parameters, such as total sleep time, sleep efficiency, sleep latency, and wake time.

### 4.3. Statistical Analyses

Initially, a descriptive analysis of the runners’ sleep–wake cycle was performed. Subsequently, time series analyses of the main sleep data and the activity–rest cycle were performed over the 35 days of the use of the actimetry, through autocorrelation using the temporal series and ARIMA (Autoregressive integrated moving average) model, with smaller normalized Bayesian Information Criterion (BIC) residual values, as classifications for selecting the most appropriate model [52].

This statistical approach provides a robust estimate of the stability and variation in biological parameters (sleep–wake cycle), allowing for the prediction or estimation of the behavior that varies with relative outcomes over the analyzed period [53]. Moreover, to our knowledge, this is the first study to continuously assess sleep and circadian rhythms in a blind trail ultramarathon runner and their specific guide. In this context, the application of this statistical method may serve as a foundation for guiding future investigations with similar populations. However, we acknowledge that descriptive analyses are more commonly used in case study designs. For this reason, such information is appropriately presented in the Supplementary Materials to enhance the understanding of individual data (Tables S7 and S8).

The quality of the adjustment was assessed by the Root Mean Square Error (RMSE), aiming to identify temporal patterns in the EFS, TTS, and WASO parameters. The significance level adopted was *p* < 0.05.

The analyses were performed using the statistical software Statistical Package for the Social Sciences (SPSS) for Windows, version 21.0 (IBM Corporation^®^, New York, NY, USA), while the graphical analyses were processed using the JAMOVI 2.3.26 software. Data are presented as mean ± standard deviation. Mean values of daily variation (∆) and percentage difference during weekdays and weekends in the pre- and post-competition period, the raw data, are available in the Supplementary Materials.

## 5. Conclusions

This is the first study to document the sleep–wake cycle patterns of a totally blind ultramarathon runner and his guide during the pre-competition, competition, and post-competition periods of an 80 km trail and mountain race. The results highlighted individual differences in the chronotypes, sleep quality, and sleep–wake cycle variability between the participants.

The blind runner exhibited a longer sleep latency, poorer subjective sleep quality, and greater irregularity in sleep–wake patterns, particularly during the post-competition period. In contrast, the guide showed greater consistency in sleep and wake times but demonstrated a higher variability in parameters such as the WASO and the number of awakenings during the same period.

These findings underscore the importance of interventions that account for individual differences in sleep management and post-competition recovery for high-performance athletes, especially those with disabilities. Future studies are needed to explore the underlying mechanisms and their impact on athletic performance and long-term health.

## 6. Limitations

As a case study with a cross-sectional design, this research presents limitations regarding the generalizability of its findings to the broader population of blind ultramarathon runners. Although it provides a detailed analysis of the sleep–wake cycle and circadian patterns of a blind athlete and their guide, these patterns cannot be assumed to be representative of other visually impaired endurance athletes.

Individual factors, such as athletic background, training strategies, chronotype, and adaptation to the guide, may influence the results, making broader comparisons challenging. Furthermore, the existing literature indicates that blind individuals exhibit variability in circadian rhythms, which can be modulated by residual light perception, compensatory strategies, and environmental conditions.

We also acknowledge the limitations related to the small sample size, intrinsic to the case study approach, as well as the 35-day monitoring window used for the time series analysis. Nevertheless, it is important to underscore that 35-day windows are higher than most actigraphy-based studies, which typically monitor sleep for 7 to 14 days. The 35-day period represented the maximum feasible duration, constrained by the memory and battery capacity of the device used. Despite these limitations, this window was sufficient to capture meaningful intra-individual variations and supported the use of time series analytical methods that account for the temporal dynamics of biological data.

Finally, future studies with larger samples and longitudinal designs, incorporating diverse analytical strategies, are essential to understanding the relationship between blindness, circadian rhythmicity, and athletic performance. Moreover, extending the data collection period will contribute to more robust and representative analyses. Such investigations may also explore targeted interventions aimed at optimizing sleep and recovery.

## Figures and Tables

**Table 1:** Time series analyses—sleep.

Variable	BTR				GTR			
	BIC	RMSE	Ljung-Box Q	*p*	BIC	RMSE	Ljung-Box Q	*p*
Bedtime (s)	20.89	32,501.63	52.15	<0.01	20.98	34,160.47	30.67	0.03
To wake up (s)	17.73	6733.56	14.93	0.66	17.09	4862.9	30.46	0.03
Time in bed (s)	17.39	5655.01	29.70	0.04	18.10	8072.47	20.06	0.33
TST (s)	17.07	4815.80	23.30	0.17	17.90	7308.73	23.83	0.16
Sleep Latency (s)	10.77	206.50	63.38	<0.01	10.54	184.79	47.87	<0.01
Sleep efficiency (%)	2.60	3.48	34.92	0.01	3.86	6.52	29.78	0.04
WASO (s)	14.50	1339.24	22.88	0.19	14.75	1511.29	24.58	0.13
Awakenings (nº)	3.26	4.84	28.36	0.05	4.74	10.10	41.04	0.002

Note: ARIMA model (0,0,0), *p* < 0.05. BTR = blind trail ultramarathon runner; GTR = guide trail ultramarathon runner; BIC = Bayesian information criterion; RMSE = Root means square error of prediction; TST = Total sleep time; WASO = Wakefulness After Sleep Onset; s = Seconds; % = Percentage; and nº = Number.

**Table 2:** Time series analyses—circadian rhythms.

Variable	BTR				GTR			
	BIC	RMSE	Ljung-Box Q	*p*	BIC	RMSE	Ljung-Box Q	*p*
M10	16.11	2998.08	216.84	0.53	16.75	4124.71	24.64	0.13
L5	14.08	1086.12	10.95	0.89	14.41	1275.71	7.97	0.97
Mesor (0.0.2)	15.49	2079.98	11.18	0.84	15.89	2674.11	10.12	0.92
Amplitude	15.74	2489.47	23.18	0.18	16.34	3353.68	20.22	0.32
Acrophase (hours)	0.22	1.06	18.13	0.44	0.31	1.11	38.53	0.003
Acrophase (radian)	19.28	14,575.13	18.13	0.44	19.36	15,203.32	38.53	0.003

Note: ARIMA model (0.0.0) or (0.0.2); *p* < 0.05. BTR = blind trail ultramarathon runner; GTR = guide trail ultramarathon runner; BIC = Bayesian information criterion; RMSE = Means squared error of the forecast; M10 = Most-active 10 h period; L5 = Mean of least active 5 h period.

## Data Availability

The data underlying this article will be shared on request to the corresponding author.

## Supplementary Materials

The following supporting information can be downloaded at https://www.mdpi.com/article/10.3390/clockssleep7020020/s1. Table S1. Sample Characterization. Table S2. Main Sleep Pattern During Monitored Weeks and Weekends. Table S3. Differences in the Five Weeks and Weekends for Main Sleep, Secondary Sleep, and Activity-Rest Cycle of the Blind Trail Ultramarathon Runner and His Guide. Table S4. Means and Percentages of Differences for Weekdays and Weekends Pre- and Post-Competition for Main Sleep of the Blind Trail Ultramarathon Runner and His Guide. Table S5. Circadian Rhythm Characteristics Monitored During Weeks and Weekends. Table S6. Means and Percent Differences in the Pre- and Post-Competition Week and Weekend for the Circadian Rhythm of the Blind Ultramarathoner and His Guide. Table S7. Secondary Sleep Pattern (Nap) During Monitored Weeks and Weekends. Table S8. Means and percentages of differences in secondary sleep during the week and weekend pre- and post-competition for the blind ultramarathoner and his guide. Figure S1. Actogram of the Blind Ultramarathon Runner. Blue rectangles represent M10. Red rectangles represent L5. Black boxes and arrows represent moments of possible phase advance. Figure S2. Actogram of the Guide Ultramarathon Runner. Blue rectangles represent M10. Red rectangles represent L5. Black boxes and arrows represent moments of possible phase advance.
