# Supplementary material for: The Sleep–Wake Cycle Pattern of a Blind Trail Ultramarathon Runner and His Guide: The World’s First Case
Source: Clocks Sleep. 2025 Apr 15;7(2):20. doi: 10.3390/clockssleep7020020 (PMC12015783; doi:10.3390/clockssleep7020020)
Supplement: Supplementary file 1 [file clockssleep-07-00020-s001.zip › clockssleep-3448216-supplementary.pdf]

Supplementary Material

Table S1. Sample Characterization.

**Characteristics**		**BTR**	**GTR**
Age (years)		54	48
Height (m)		1.75	1.69
Body Mass (kg)		80.0	69.0
BMI (kg/m²)		26.1	24.2
**MEQ**	Classification	Morning	Intermediate
	Score	63	52
**ESS**	Classification	High chance	High chance
	Score	7	10
**ISI**	Classification	Mild insomnia	Insignificant insomnia
	Score	11	1
**PSQI**	Sleep Quality	Poor	Good
	Sleep Latency (min)	60	10
	Total Sleep Time (min)	480	410
	Sleep Efficiency (%)	88.9	97.6
	Score	5	3

Note: Data are presented as absolute values (n). BTR = Blind trail ultramarathon runner; GTR = Guide trail ultramarathon runner; MEQ = Morningness-Eveningness Questionnaire; ESS = Epworth Sleepiness Scale; ISI = Insomnia Severity Index; PSQI = Pittsburgh Sleep Quality Index; m = meters; kg = kilograms; kg/m² = kilograms per square meter; min = minutes; % = percentage.

Table S2. Main Sleep Pattern During Monitored Weeks and Weekends.

	Variables	BTR Weeks					GTR Weeks				
		1st	2st	3st	4st (pre)	5st (post)	1st	2st	3st	4st (pre)	5st (post)
Weekdays - Main Sleep	Bedtime (h:min)	22:54 (00:48)	22:48 (00:40)	23:42 (00:16)	01:04 (01:16)	00:39 (05:19)	23:26 (00:48)	23:34 (00:14)	23:28 (00:14)	23:26 (03:33)	22:18 (01:26)
	Wake-up time (h:min)	05:47 (01:43)	06:23 (00:30)	06:30 (00:39)	06:53 (01:11)	07:59 (03:25)	06:24 (00:14)	06:40 (00:02)	06:34 (00:19)	06:42 (00:46)	06:14 (00:15)
	Time in bed (h:min)	06:53 (01:36)	07:34 (00:15)	06:47 (00:34)	05:49 (01:29)	07:19 (02:06)	06:58 (00:59)	07:06 (00:14)	07:05 (00:20)	07:16 (03:47)	07:55 (01:39)
	TST (h:min)	06:15 (01:32)	06:46 (00:20)	05:59 (00:33)	05:10 (01:15)	06:10 (01:37)	06:20 (00:47)	06:04 (00:10)	06:11 (00:16)	06:17 (03:21)	07:01 (01:21)
	Sleep latency (h:min)	00:04 (00:02)	00:04 (00:01)	00:06 (00:02)	00:03 (00:00)	00:05 (00:04)	00:03 (00:02)	00:17 (00:07)	00:04 (00:02)	00:04 (00:02)	00:03 (00:02)
	Sleep efficiency (%)	90.52 (2.00)	89.48 (2.76)	88.29 (1.51)	89.17 (2.15)	84.84 (2.94)	90.61 (9.67)	85.61 (2.44)	87.35 (1.96)	89.01 (7.90)	89.07 (6.09)
	WASO (h:min)	00:27 (00:07)	00:41 (00:11)	00:36 (00:06)	00:34 (00:17)	01:00 (00:32)	00:22 (00:43)	00:29 (00:08)	00:46 (00:12)	00:50 (00:32)	00:49 (00:26)
	Nº of awakenings (n)	12.00 (4.74)	14.25 (3.90)	12.75 (3.16)	12.00 (5.10)	15.40 (6.15)	13.33 (7.63)	18.75 (3.00)	23.00 (7.00)	13.50 (11.77)	18.40 (12.19)
Weekends - Main Sleep	Bedtime (h:min)	01:44 (04:14)	23:17 (00:56)	22:44 (01:39)	23:39 (00:49)	01:39 (00:00)	02:31 (04:26)	01:48 (01:17)	23:29 (00:37)	00:17 (00:22)	03:09 (05:03)
	Wake-up time (h:min)	07:15 (02:44)	06:37 (00:15)	04:42 (03:08)	07:19 (00:39)	07:00 (00:00)	07:51 (01:26)	07:22 (00:55)	05:24 (00:44)	06:40 (00:09)	07:28 (06:08)
	Time in bed (h:min)	05:31 (01:30)	07:19 (00:43)	05:58 (02:49)	07:40 (00:32)	05:21 (00:00)	05:20 (03:00)	05:33 (01:31)	05:55 (01:09)	06:23 (00:26)	04:19 (02:31)
	TST (h:min)	04:49 (01:24)	06:07 (00:27)	05:12 (02:18)	06:58 (00:25)	04:32 (00:00)	05:06 (02:57)	04:38 (01:40)	05:06 (00:58)	05:46 (00:15)	03:18 (01:57)
	Sleep latency (h:min)	00:06	00:03	00:03	00:03	00:04	00:01	00:07	00:05	00:06	00:10

	(00:02)	(00:01)	(00:02)	(00:03)	(00:00)	(00:00)	(00:01)	(00:02)	(00:02)	(00:08)
Sleep efficiency (%)	86.84<br>(1.78)	83.97<br>(3.97)	88.64<br>(3.27)	90.86<br>(0.82)	84.74<br>(0.00)	94.47<br>(2.33)	80.86<br>(9.73)	86.80<br>(7.52)	90.44<br>(2.73)	79.11<br>(7.66)
WASO (h:min)	00:34<br>(00:08)	01:03<br>(00:23)	00:39<br>(00:31)	00:35<br>(00:03)	00:44<br>(00:00)	00:12<br>(00:02)	00:30<br>(00:12)	00:41<br>(00:31)	00:25<br>(00:15)	00:48<br>(00:33)
Nº of awakenings (n)	11.00<br>(2.50)	16.33<br>(9.67)	13.25<br>(7.73)	14.67<br>(2.62)	12.00<br>(0.00)	7.00<br>(4.50)	10.33<br>(3.86)	15.33<br>(6.98)	16.67<br>(5.56)	12.00<br>(7.07)

Note: Data are presented as mean ± standard deviation (SD). BTR = Blind trail ultramarathon runner; GTR = Guide trail ultramarathon runner; Pre = pre-race; Post = post-race; TST = total sleep time; WASO = wake after sleep onset; h = hours; min = minutes; % = percentage; Nº = number.

Table S3. Differences in the Five Weeks and Weekends for Main Sleep. Secondary Sleep. and Activity-Rest Cycle of the Blind trail ultramarathon runner and his Guide.

	Variables	**BTR** **Weekdays** (mean±SD)	**GTR** **Weekdays** (mean±SD)	**Δ**	**%**	**BTR** **Weekends** (mean±SD)	**GTR** **Weekends** (mean±SD)	**Δ**	**%**
**Main Sleep**	Time in bed (h:min)	06:52 (00:40)	07:16 (00:22)	00:24	5.50	06:22 (01:03)	05:30 (00:46)	00:52	13.60
	TST (h:min)	06:04 (00:35)	06:23 (00:22)	00:20	5.00	05:31 (01:00)	04:47 (00:55)	00:44	13.30
	Sleep latency (h:min)	00:04 (00:01)	00:06 (00:05)	00:02	33.33	00:04 (00:01)	00:05 (00:03)	00:01	20.00
	Sleep efficiency (%)	88.46 (2.17)	88.33 (1.90)	0.13	0.15	87.01 (2.82)	86.33 (6.43)	0.68	0.80
	WASO (h:min)	00:40 (00:12)	00:39 (00:12)	00:01	2.50	00:43 (00:11)	00:31 (00:14)	00:12	27.90
	Nº of awakenings (n)	13.28 (1.49)	17.39 (4.06)	4.11	23.63	13.45 (2.11)	12.26 (3.88)	1.19	8.85
**Secondary Sleep**	Time in bed (h:min)	00:47 (00:12)	01:09 (00:41)	00:22	31.90	01:49 (01:12)	01:40 (01:10)	09:00	8.30
	TST (h:min)	00:35 (00:07)	00:59 (00:37)	00:24	40.70	01:30 (01:05)	00:49 (00:54)	41:00	45.60
	Sleep latency (h:min)	00:03 (00:00)	00:03 (00:01)	00:00	0.00	00:04 (00:01)	00:01 (00:01)	03:00	75.00
	Sleep efficiency (%)	75,40 (4.02)	83,52 (7.63)	8.12	9.72	82,01 (12.02)	77,99 (21.18)	4.02	4.90
	WASO (h:min)	00:07 (00:05)	00:05 (00:04)	00:02	28.60	00:13 (00:17)	00:09 (00:13)	00:04	30.80
	Nº of awakenings (n)	2,11 (0.89)	1,50 (1.29)	0.61	28.90	3,10 (2.40)	2,75 (4.27)	0.35	11.30

Note: Data are expressed as mean standard deviation (SD), absolute difference (Δ) and percentage difference (%). BTR = Blind trail ultramarathon runner; GTR = Guide trail ultramarathon runner; TST = total sleep time; WASO = wake after sleep onset; h = hours; min = minutes; % = percentage; Nº = number. M10 = most-active 10-hour period; L5 = mean of least-active 5-hour period; n = number.

Table S4. Means and Percentages of Differences for Weekdays and Weekends Pre- and Post-Competition for Main Sleep of the Blind trail ultramarathon runner and his Guide.

	**Variables**	**BTR Pre-Week**	**GTR Pre-Week**	**Δ**	**%**	**BTR Post-Week**	**GTR Post-Week**	**Δ**	**%**
**Weekdays – Main Sleep**	Time in bed (h:min)	05:49	07:16	01:27	20.00	07:55	07:19	00:36	7.60
	TST (h:min)	05:10	06:17	01:07	17.80	07:01	06:10	00:51	12.10
	Sleep latency (h:min)	00:03	00:04	00:01	25.00	00:03	00:05	00:02	40.00
	Sleep efficiency (%)	00:34	00:50	00:16	32.00	00:49	01:00	00:11	18.30
	WASO (h:min)	89.17	89.01	0.16	0.18	89.07	84.84	4.23	4.75
	Nº of awakenings (n)	12.00	13.50	1.50	11.10	18.40	15.40	3.00	16.30
**Weekends – Main Sleep**	Time in bed (h:min)	07:40	06:23	01:17	16.74	04:19	05:21	01:02	19.30
	TST (h:min)	06:58	05:46	01:12	17.20	03:18	04:32	01:14	27.20
	Sleep latency (h:min)	00:03	00:06	00:03	50.00	00:10	00:04	00:06	60.00
	Sleep efficiency (%)	00:35	00:25	00:10	28.60	00:48	00:44	00:04	8.33
	WASO (h:min)	90.86	90.44	0.42	0.50	79.11	84.74	5.63	6.64
	Nº of awakenings (n)	14.67	16.67	2.00	12.00	12.00	12.00	0.00	0.00

Note:Data are expressed as means, absolute difference (Δ) and percentage difference (%). BTR = Blind trail ultramarathon runner; GTR = Guide trail ultramarathon runner; TST = Total Sleep Time; WASO = Wake After Sleep Onset; h = hours; min = minutes; % = percentage; nº = number.

Table S5. Circadian Rhythms Characteristics Monitored During Weeks and Weekends.

	Variables	BTR Weeks 1st	BTR Weeks 2st	BTR Weeks 3st	BTR Weeks 4st (pre)	BTR Weeks 5st (post)	GTR Weeks 1st	GTR Weeks 2st	GTR Weeks 3st	GTR Weeks 4st (pre)	GTR Weeks 5st (post)
**Weekdays – Activity-Rest Cycle**	M10 (counts)	6315.92 (4105.87)	5039.38 (3716.26)	7164.62 (3545.88)	6352.52 (3060.50)	3566.70 (524.87)	5770.98 (4657.63)	5696.68 (4249.39)	3879.96 (3204.99)	8276.36 (3347.69)	2016.99 (1061.77)
	L5 (counts)	557.48 (688.08)	666.54 (1135.09)	543.56 (871.11)	775.35 (763.78)	213.91 (188.17)	317.07 (490.98)	327.94 (445.38)	289.86 (343.05)	1915.72 (2509.07)	657.75 (1177.19)
	Peak 1	1440	1207	1440	1000	1493	1437	1440	1074	1013	1434
	Peak 2	1575	1383	2120	1393	1715	1792	2096	1405	1609	1664
	Peak 3	1753	2366	2353	1920	2305	2148	2217	2194	2027	2157
	Mesor (counts)	3239.91	3137.44	3754.05	4352.09	2184.49	3068.45	3128.63	2211.73	5667.39	1504.54
	Amplitude (counts)	2160.43	2694.92	3634.58	2074.82	1451.89	2805.21	3137.89	1441.40	3025.78	790.91
	Acrophase (hours)	17:06	16:59	17:59	13:02	14:06	18:45	18:17	17:31	13:04	12:13
	Acrophase (radians)	-4.4796	-4.4477	-4.7088	-3.4132	-3.6935	-4.9111	-4.7907	-4.5866	-3.4210	-3.2023
**Weekends – Activity-Rest Cycle**	M10 (counts)	7237.80 (0.00)	2550.47 (269.58)	9041.79 (4248.47)	9180.47 (5717.73)	6594.98 (260.56)	9099.40 (0.00)	3142.42 (748.72)	10369.87 (5380.69)	10485.70 (6354.68)	8523.49 (1009.87)
	L5 (counts)	889.33 (0.00)	92.25 (3.65)	363.89 (258.23)	88.49 (0.58)	935.37 (820.52)	2864.16 (0.00)	537.97 (452.62)	296.17 (117.70)	91.95 (5.76)	351.01 (29.24)
	Peak 1	143	776	-	-	892	250	817	-	-	718
	Peak 2	291	1274	977	738	922	344	941	728	548	826
	Peak 3	503	1397	1407	1422	966	488	1297	1438	823	1022
	Mesor (counts)	5325.54	1431.42	3870.11	4238.11	4022.48	7106.74	1803.18	4963.63	4609.88	6069.34
	Amplitude (counts)	4996.15	1198.69	5230.06	4890.19	694.97	4035.08	1335.76	6137.16	5825.21	653.58
	Acrophase (hours)	21:56	11:55	10:57	11:17	02:00	20:46	15:22	10:03	10:48	16:51
	Acrophase (radians)	-5.7461	-3.1233	-2.8686	-2.9582	-0.5245	-5.4367	-4.0255	-2.6323	-2.8289	-4.4155

Note: Data expressed as absolute values (n), means, and standard deviation (SD). BTR = blind trail ultramarathoner; GTR = guide trail ultramarathoner; Pre = pre-race; Post = post-race; M10 = Most-active 10-hour period (mean activity counts); L5 = Least-active 5-hour period (mean activity counts). Peak 1, Peak 2, Peak 3: Three highest peaks of recorded activity counts. Mesor: Mean of the circadian rhythm (activity counts). Amplitude: Difference between the maximum

and minimum values of the circadian rhythms (activity counts). Acrophase (hours/radians): Time and phase of the average activity peak during the circadian cycle.

Table S6. Means and Percent Differences of the Pre- and Post-Competition Week and Weekend for the Circadian Rhythms of the Blind Ultramarathoner and His Guide.

	Variables	BTR Pre-Week	GTR Pre-Week	Δ	%	BTR Post-Week	GTR Post-Week	Δ	%
**Weekdays – Activity-Rest Cycle**	M10 (counts)	6352.52	8276.36	1923.84	23.25	3566.7	2016.99	1549.71	43.45
	L5 (counts)	775.35	1915.72	1140.37	59.53	213.91	657.75	443.84	67.48
	Peak 1	1000.00	1013.00	13.00	1.28	1493.00	1434.00	59.00	3.95
	Peak 2	1393.00	1609.00	216.00	13.42	1715.00	1664.00	51.00	2.97
	Peak 3	1920.00	2027.00	107.00	5.28	2305.00	2157.00	148.00	6.42
	Mesor (counts)	4352.09	5667.39	1315.3	23.21	2184.49	1504.54	679.95	31.13
	Amplitude (counts)	2074.82	3025.78	950.96	31.43	1451.89	790.91	660.98	45.53
	Acrophase (hours)	13:02	13:04	00:02	0.26	14:06	12:13	01:53	13.36
	Acrophase (radians)	-3.4132	-3.421	0.0078	0.23	-3.6935	-3.2023	0.4912	13.30
**Weekends – Activity-Rest Cycle**	M10 (counts)	9180.47	10485.7	1305.23	12.45	6594.98	8523.49	1928.51	22.63
	L5 (counts)	88.49	91.95	3.46	3.76	935.37	351.01	584.36	62.47
	Peak 1		-	-	-	892	718	174	19.51
	Peak 2	738.00	548.00	190.00	25.75	922.00	826.00	96.00	10.41
	Peak 3	1422.00	823.00	599.00	42.12	966.00	1022.00	56.00	5.48
	Mesor (counts)	4238.11	4609.88	371.77	8.06	4022.48	6069.34	2046.86	33.72
	Amplitude (counts)	4890.19	5825.21	935.02	16.05	694.97	653.58	41.39	5.96
	Acrophase (hours)	11:17	10:48	00:29	4.28	02:00	16:51	14:51:00	88.13
	Acrophase (radians)	-2.9582	-2.8289	0.1293	4.37	-0.5245	-4.4155	3.891	88.12

Note:Data expressed as absolute values (n), means, and standard deviation (SD). BTR = blind trail ultramarathoner; GTR = guide trail ultramarathoner; Pre = pre-race; Post = post-race; M10 = Most-active 10-hour period (mean activity counts); L5 = Least-active 5-hour period (mean activity counts). Peak 1, Peak 2, Peak 3: Three highest peaks of recorded activity counts. Mesor: Mean of the circadian rhythm (activity counts). Amplitude: Difference between the maximum and minimum values of the circadian rhythms (activity counts). Acrophase (hours/radians): Time and phase of the average activity peak during the circadian cycle.

Table S7. Secondary Sleep Pattern (Nap) During Monitored Weeks and Weekends.

	**Variables**	**BTR Weeks**					**GTR Weeks**				
		**1st**	**2st**	**3st**	**4st (pre)**	**5st (post)**	**1st**	**2st**	**3st**	**4st (pre)**	**5st (post)**
**Weekdays – Secondary Sleep**	Bedtime (h:min)	17:46 (02:44)	12:51 (00:00)	14:40 (00:30)	23:50 (00:00)	15:10 (01:39)	20:57 (00:30)	20:57 (00:00)	no sleep	14:52 (00:00)	09:02 (00:00)
	Wake-up time (h:min)	18:23 (02:47)	13:29 (00:00)	15:27 (00:43)	00:37 (00:00)	16:18 (02:13)	21:34 (00:05)	21:32 (00:00)	no sleep	16:17 (00:00)	11:03 (00:00)
	Time in bed (h:min)	00:37 (00:04)	00:38 (00:00)	00:47 (00:13)	00:47 (00:00)	01:08 (00:34)	00:37 (00:25)	00:35 (00:00)	no sleep	01:25 (00:00)	02:01 (00:00)
	TST (h:min)	00:28 (00:03)	00:29 (00:00)	00:34 (00:12)	00:38 (00:00)	00:47 (00:20)	00:26 (00:12)	00:31 (00:00)	no sleep	01:12 (00:00)	01:47 (00:00)
	Sleep latency (h:min)	00:04 (00:01)	00:03 (00:00)	00:03 (00:01)	00:04 (00:00)	00:04 (00:01)	00:04 (00:01)	00:03 (00:00)	no sleep	00:03 (00:00)	00:05 (00:00)
	Sleep efficiency (%)	76.91 (2.63)	76.32 (0.00)	70.69 (5.98)	80.85 (0.00)	72.27 (10.55)	72.40 (0.50)	88.57 (0.00)	no sleep	84.71 (0.00)	88.43 (0.00)
	WASO (h:min)	00:03 (00:03)	00:05 (00:00)	00:08 (00:02)	00:04 (00:00)	00:15 (00:16)	00:03 (00:11)	00:00 (00:00)	no sleep	00:09 (00:00)	00:08 (00:00)
	Nº of awakenings (n)	1.33 (1.25)	1.00 (0.00)	2.50 (1.50)	3.00 (0.00)	2.75 (2.77)	1.00 (0.50)	0.00 (0.00)	no sleep	3.00 (0.00)	2.00 (0.00)
**Weekends – Secondary Sleep**	Bedtime (h:min)	16:29 (00:00)	23:48 (09:35)	13:28 (00:59)	21:08 (00:00)	18:52 (00:00)	14:58 (00:00)	18:52 (03:21)	18:41 (00:00)	no sleep	13:26 (00:00)
	Wake-up time (h:min)	20:11 (00:00)	01:13 (09:05)	15:40 (00:57)	22:25 (00:00)	19:24 (00:00)	15:23 (00:00)	21:42 (02:18)	19:05 (00:00)	no sleep	14:03 (00:00)
	Time in bed (h:min)	03:42 (00:00)	01:25 (00:30)	02:12 (00:02)	01:17 (00:00)	00:32 (00:00)	00:25 (00:00)	02:50 (01:03)	00:24 (00:00)	no sleep	00:37 (00:00)
	TST (h:min)	03:22 (00:00)	01:11 (00:28)	01:21 (00:20)	01:10 (00:00)	00:27 (00:00)	00:25 (00:00)	02:10 (00:52)	00:12 (00:00)	no sleep	00:32 (00:00)

Sleep latency (h:min)	00:06 (00:00)	00:04 (00:01)	00:05 (00:01)	00:03 (00:00)	00:04 (00:00)	00:00 (00:00)	00:03 (00:00)	00:03 (00:00)	no sleep	00:04 (00:00)
Sleep efficiency (%)	90.99 (0.00)	82.13 (3.95)	61.68 (15.42)	90.91 (0.00)	84.98 (0.00)	100.00 (0.00)	75.50 (2.61)	50.00 (0.00)	no sleep	86.49 (0.00)
WASO (h:min)	00:11 (00:00)	00:08 (00:04)	00:43 (00:23)	00:03 (00:00)	00:00 (00:00)	00:00 (00:00)	00:28 (00:07)	00:08 (00:00)	no sleep	00:00 (00:00)
Nº of awakenings (n)	6.00 (0.00)	2.50 (0.50)	5.00 (0.00)	2.00 (0.00)	0.00 (0.00)	0.00 (0.00)	9.00 (0.50)	2.00 (0.00)	no sleep	0.00 (0.00)

Note: Data expressed as means and standard deviation (SD). BTR = Blind trail ultramarathon runner; GTR = Guide trail ultramarathon runner; Pre = pre-race; Post = post-race; TST = total sleep time; WASO = wake after sleep onset; h = hours; min = minutes; % = percentage; n = number.

Table S8. Means and percentages of differences in secondary sleep during the week and weekend pre- and post-competition for the blind ultramarathoner and his guide.

	Variables	BTR Pre-Week	GTR Pre-Week	Δ	%	BTR Post-Week	GTR Post-Week	Δ	%
Weekdays – Secondary Sleep	Time in bed (h:min)	00:47	01:25	00:38	44.71	01:08	02:01	00:53	43.80
	TST (h:min)	00:38	01:12	00:34	47.22	00:47	01:47	01:00	56.07
	Sleep latency (h:min)	00:04	00:03	00:01	25.00	00:04	00:05	00:01	20.00
	Sleep efficiency (%)	00:04	00:09	00:05	55.56	00:15	00:08	00:07	46.67
	WASO (h:min)	80.85	84.71	3.86	4.56	72.27	88.43	16.16	18.27
	Nº of awakenings (n)	3.00	3.00	0.00	0.00	2.75	2.00	0.75	27.27
Weekends – Secondary Sleep	Time in bed (h:min)	01:17	00:00	01:17	100.00	00:32	00:37	00:05	13.51
	TST (h:min)	01:10	00:00	01:10	100.00	00:27	00:32	00:05	15.63
	Sleep latency (h:min)	00:03	00:00	00:03	100.00	00:04	00:04	00:00	0.00
	Sleep efficiency (%)	00:03	00:00	00:03	100.00	00:00	00:00	00:00	-
	WASO (h:min)	90.91	0.00	90.91	100.00	84.98	86.49	1.51	1.75
	Nº of awakenings (n)	2.00	0.00	2.00	100.00	0.00	0.00	0.00	-

Note: Data are expressed as mean. standard deviation (SD), absolute difference (Δ) and percentage difference (%). BTR = blind ultramarathoner; GTR = guide ultramarathoner; TST = total sleep time; WASO = wake after sleep onset; h = hours; min = minutes; % = percentage; nº = number.

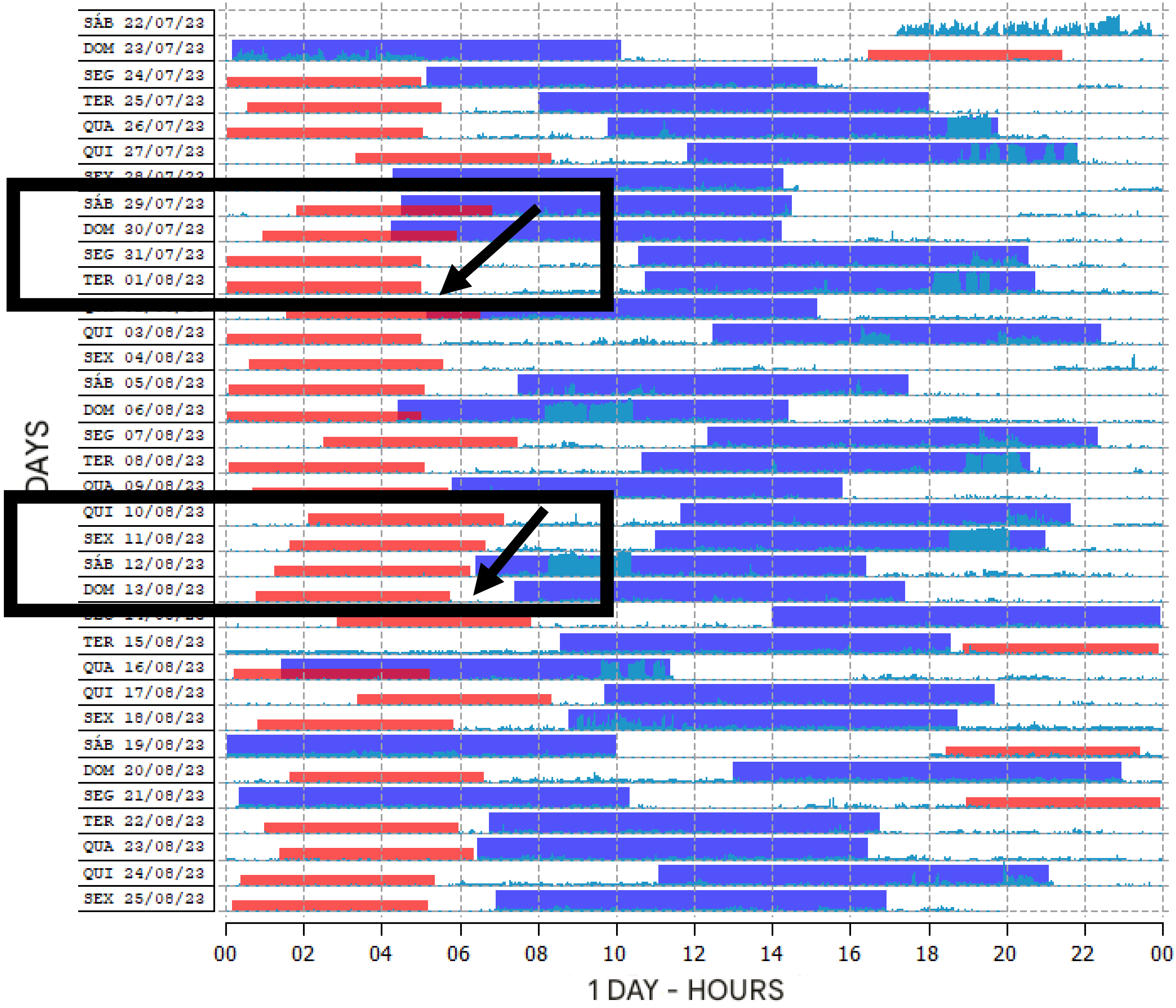

Figure S1. Actogram of the Blind Ultramarathon Runner. Blue rectangles represent M10. Red rectangles represent L5. Black boxes and arrows represent moments of possible phase advance.

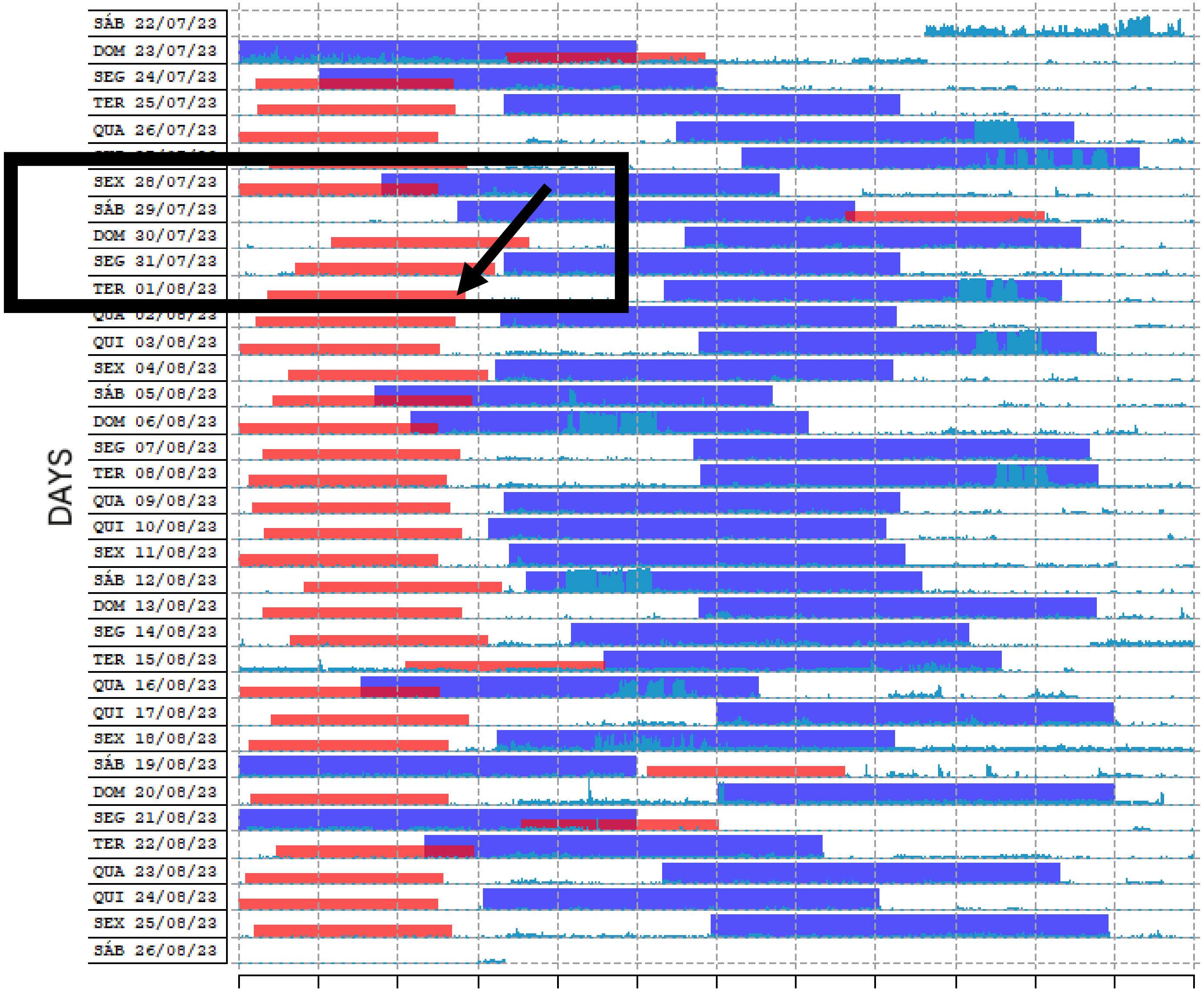

Figure S2. Actogram of the Guide Ultramarathon Runner. Blue rectangles represent M10. Red rectangles represent L5. Black boxes and arrows represent moments of possible phase advance.
